# Simplicity at the cost of predictive accuracy in diffuse large B‐cell lymphoma: a critical assessment of the R‐IPI, IPI, and NCCN‐IPI

**DOI:** 10.1002/cam4.1271

**Published:** 2017-12-13

**Authors:** Jorne Biccler, Sandra Eloranta, Peter de Nully Brown, Henrik Frederiksen, Mats Jerkeman, Karin E. Smedby, Martin Bøgsted, Tarec C. El‐Galaly

**Affiliations:** ^1^ Department of Hematology Aalborg University Hospital Aalborg Denmark; ^2^ Department of Clinical Medicine Aalborg University Aalborg Denmark; ^3^ Department of Medicine Solna Clinical Epidemiology Unit Karolinska Institutet Stockholm Sweden; ^4^ Department of Hematology Copenhagen University Hospital Copenhagen Denmark; ^5^ Department of Hematology Odense University Hospital Odense Denmark; ^6^ Department of Oncology Lund University Lund Sweden; ^7^ Hematology Center Karolinska University Hospital Solna Sweden

**Keywords:** Diffuse large B‐cell lymphoma, IPI, prognosis, prognostic factors, risk modeling

## Abstract

The international prognostic index (IPI) and similar models form the cornerstone of clinical assessment in newly diagnosed diffuse large B‐cell lymphoma (DLBCL). While being simple and convenient to use, their inadequate use of the available clinical data is a major weakness. In this study, we compared performance of the International Prognostic Index (IPI) and its variations (R‐IPI and NCCN‐IPI) to a Cox proportional hazards (CPH) model using the same covariates in nondichotomized form. All models were tested in 4863 newly diagnosed DLBCL patients from population‐based Nordic registers. The CPH model led to a substantial increase in predictive accuracy as compared to conventional prognostic scores when evaluated by the area under the curve and other relevant tests. Furthermore, the generation of patient‐specific survival curves rather than assigning patients to one of few predefined risk groups is a relevant step toward personalized management and treatment. A test‐version is available on lymphomapredictor.org.

## Introduction

Diffuse large B‐cell lymphoma (DLBCL) is the most common type of lymphoma in the Western World 1. Despite improved treatment with immunochemotherapy, 30% of the patients are refractory to treatment or relapse after initial response to treatment 2. The variable outcomes of DLBCL could, at least partially, be explained by the genetic heterogeneity of DLBCL, which is increasingly recognized in the 2016 revision of the World Health Organization classification of lymphoid neoplasms 3. In particular, cell of origin (activated B‐cell [ABC] versus germinal center B‐cell [GCB] phenotype and chromosomal translocations involving MYC and BCL2 or/and BCL6 (double/triple hit) are considered highly relevant for prognosis 3. The differences in the molecular genetics of the ABC and GCB subtypes of DLBCL provide rationale for differential use of novel drugs and may be exploited in future DLBCL treatment 4. However, despite advances in the understanding of molecular genetics of DLBCL, R‐CHOP (rituximab, cyclophosphamide, doxorubicin, vincristine, and prednisolone) or similar therapies remain the standard treatment for DLBCL and clinical prognostic scores still weigh heavily in treatment decisions 5. The international prognostic index (IPI), developed almost 25 years ago, was the first widely accepted prognostic score for aggressive lymphomas. The IPI sums a number of individual risk factors (lactate dehydrogenase [LDH] >upper normal limit, age at diagnosis >60 years, Ann Arbor stage III or IV disease, Eastern Oncology Cooperative Group [ECOG] performance score >1, and >1 extranodal site) with equal weight in a total score ranging from 0 to 5. Patients are then assigned to four prognostic subgroups with different outcomes: low (score 0–1), intermediate‐low (2), intermediate‐high (3), and high‐risk disease (4–5) 6. Population‐based studies and clinical trial data have confirmed the robustness of the IPI despite treatment changes over time 2, 7. Modified versions of the IPI with fewer prognostic subgroups or more variables have been proposed over the years 7, 8, 9. Simplicity of clinical prognostic scores was required for use at the time they were developed, but more complex prognostic models with better utilization of data are relevant alternatives with today's widespread availability of mobile devices. Previous studies in medicine have shown that dichotomizing continuous variables leads to a substantial loss of model performance 10, 11. In this study, we show how outcome prediction in DLBCL is substantially improved by a Cox proportional hazards (CPH) model assuming linearity for continuous covariates.

## Methods

### Patients

This study was based on the nationwide Danish (LYFO) and Swedish (SLR) lymphoma registers, which contain detailed information on baseline clinicopathologic features, treatment, and outcome of lymphoma patients in both countries. As a rule, variables are entered prospectively by local hematology departments and the coverage relative to the national pathology or cancer registers is 98% for LYFO and 95% for SLR 12, 13. Both registers are periodically merged with the national civil registers, which contain the dates of death of all deceased inhabitants. Reporting of relapse/progression to LYFO is mandatory and safeguarding measures against missing registrations include merging with the national Danish Pathology and the National Danish Patient Registries (which contains information on hospital reimbursements).

The inclusion criteria in this study were (1) newly diagnosed DLBCL between 1 January 2006 and 31 December 2014 for the Danish cohort and between 01 July 2007 and 31 December 2013 for the Swedish cohort (2) no CNS (parenchymal, leptomeningeal, and/or intravitreal) involvement at baseline, (3) full treatment information and treated with R‐CHOP, R‐CHOEP, or R‐CEOP therapy, (4) age ≥ 18 years, and (5) full information on all IPI variables and LDH levels. Inclusion criterion (1) was necessary to ensure the availability of countrywide LDH reference levels.

### Prognostic models

We compared the model with continuous variables to the IPI, Revised IPI (R‐IPI), and National Comprehensive Cancer Network IPI (NCCN‐IPI). The R‐IPI was developed by analyzing rituximab‐treated patients from British Columbia (Canada) and consists of a regrouping of the IPI‐score into only three risk groups: low (0), intermediate (1–2), and high risk (3–5) 7. A more recent and refined IPI variation, the NCCN‐IPI, introduced incremental scores for increasing age and normalized LDH ratio (measured LDH divided by the upper normal level). To accomplish this, the cut‐points for the effect of age at diagnosis and normalized LDH ratio were chosen based on a Cox proportional hazards (CPH) model with cubic spline effects 9. This led to the categorization of age into the following categories: <=40 (score 0), 41–60 (1), 61–75 (2), and >75 (3). The normalized LDH ratio was categorized as ≤1 (0), 1–3 (1), and >3 (2). Furthermore, involvement of more than one extranodal site as a risk factor was replaced by the involvement of at least one of the following sites: lungs, bone marrow, liver/gastrointestinal tract, and/or central nervous system. The dichotomization of the Ann Arbor stage and ECOG performance status remained as in the IPI. The resultant NCCN‐IPI score ranges from 0 to 8 and patients are categorized into the risk groups low (0–1), low‐intermediate (2–3), high‐intermediate (4–5), and high (6–8) 9.

### Statistics

The overall survival (OS) was measured as the time from diagnosis until death from any cause. Patients still alive in May 2016 (LYFO) and April 2015 (SLR) were censored. The regression model under investigation was a CPH model including the same variables as the IPI. Logarithmic transformation of the normalized LDH ratio was performed to avoid overly large and influential values. The effects of age, normalized LDH ratio, and number of extranodal sites were modeled as continuous linear effects, whereas ECOG performance and Ann Arbor stage were included as categorical. Country‐specific survival curves according to the IPI, R‐IPI, and NCCN‐IPI were obtained using the Kaplan–Meier estimator. Finally, to inspect loss of information caused by variable dichotomizing in the IPI, five additional Cox models, each with one dichotomized variable, were tested.

Comparisons of different prognostic models were performed by estimating different performance measures by repeated 10‐fold cross validation in the Danish cohort. Furthermore, to test the generalizability of the model obtained from the Danish cohort, the Swedish cohort was used as an external validation set. Overall model performance of the different models was summarized using the time‐varying area under the curve (AUC), the C‐index, and the integrated Brier score (IBS). The AUC at time t summarizes how well the risk score discriminates between being alive, *Y*(i) = 1, or dead, *Y*(*t*) = 0, at time *t* 14. The C‐index summarizes the time‐varying AUC over time. It measures the probability of a patient having a higher risk score than a second patient given that the first patient died earlier 15. The Brier score at time t measures the squared prediction error between the predicted probabilities, *S*(*t*), of being alive at time *t* and the observed status of the patients at time *t*, that is, (*Y*(*t*)–*S*(*t*))^2 16, 17. The IBS is the integral of the Brier score over a prespecified time interval and summarizes the predictive power of the survival model in this interval. For the IBS and the C‐index, a cut‐off point of 7.5 years was used. To measure the capability of the CPH model to identify high‐risk patients, we explored whether the CPH model could identify patients with a predicted 2‐year survival of less than 50%, as models capable of this have been called for and represents an unmet need in prognostic modeling 9. The positive predictive values (PPV) are then an estimate of the proportion of deaths in the high‐risk group 18. Calculations were performed in R version 3.4.1 19. The IBSs were obtained using the “pec” package version 2.5.3, the C‐indices from the “survC1” package version 1.0‐2, and the time‐varying AUCs, and PPVs from the “timeROC” package version 0.3 20, 21, 22.

## Results

In total, 2696 and 2167 DLBCL patients were included from the LYFO and the SLR, respectively. Relative to the population sizes, fewer Swedish patients than expected were included as a result of missing information on critical baseline characteristics and treatment information in the surveyed period (Fig. S1, flow chart). Summaries of patient characteristics are shown in Table 1. Patients included from the SLR generally had lower Ann Arbor stage, fewer extranodal sites involved, and a higher frequency of elevated LDH as compared to Danish patients. Differences in extranodal site involvement may be explained by structural differences in the registers, that is, more anatomic sites are by default registered in the Danish register. Differences in the frequencies of Ann Arbor stages may be explained by the use of Musshoff stage in the SLR, which may represent cases that would have been diagnosed as stage IV in LYFO.

**Table 1:** Clinical characteristics of Swedish and Danish patients with diffuse large cell B‐cell lymphoma

	Denmark (*n* = 2696)	Sweden (*n* = 2167)
Median age, years (range)	67 (18–95)	67 (18–95)
Sex ratio (M/F)	1.29	1.33
Ann Arbor stage, *n* (%)		
I	527 (19.6)	443 (20.4)
II	481 (17.8)	586 (27.0)
III	587 (21.8)	405 (18.6)
IV	1101 (40.8)	733 (33.8)
ECOG performance status, *n* (%)		
0	1320 (49.0)	1068 (49.2)
I	921 (34.2)	758 (35.0)
II	249 (9.2)	188 (8.7)
III	143 (5.3)	122 (5.6)
IV	63 (2.3)	31 (1.4)
Median number of extranodal sites, (range)^a^	1 (0–10)	0 (0–6)
Normalized LDH		
>1, *n* (%)	1473 (54.6)	1309 (60.4)
Median	1.06	1.14

a The Danish lymphoma registry includes more extranodal sites than the Swedish Lymphoma Registry.

**Figure 1:**
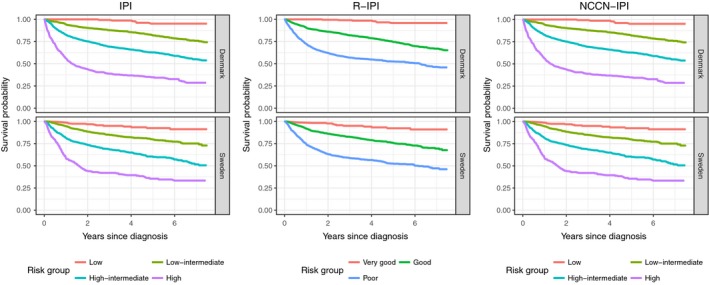
Estimated survival probability over the first 7.5 years postdiagnosis shown as Kaplan–Meier curves for the different risk groups defined by the International Prognostic Index (IPI), Revised IPI (R‐IPI), and National Comprehensive Cancer Network IPI (NCCN‐IPI). The estimates from the Danish and Swedish cohorts are shown in the upper and lower plots, respectively.

The median follow‐up was 5.5 years in the Danish cohort and 4.8 years in the Swedish cohort. The 5‐year OS probabilities among Danish and Swedish patients were 0.67 (0.65; 0.69) and 0.68 (0.66; 0.70), respectively. The NCCN‐IPI, R‐IPI, and IPI specific OS curves were estimated using the Kaplan–Meier method (Fig. 1). The NCCN‐IPI identified a group of patients with a 2‐year OS <50%, but this was not the case for the IPI and R‐IPI (Fig. 1).

**Figure 2:**
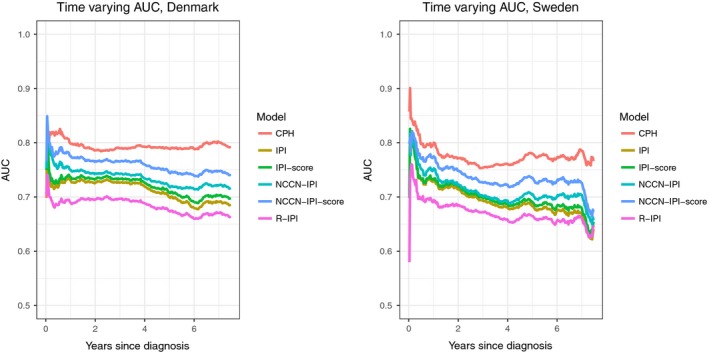
Time‐varying area under the curve (AUC) of Cox proportional hazards (CPH) model and the International Prognostic Index (IPI) grouping, IPI score, National Comprehensive Cancer Network IPI (NCCN‐IPI) grouping, NCCN‐IPI score, and Revised IPI (R‐IPI) grouping, combined with the Kaplan–Meier estimator. These were estimated using repeated 10‐fold cross validation in the Danish cohort, and the Swedish cohort was used as validation data.

The hazard ratios (HR) from the CPH model obtained from the Danish data are shown in Table 2. When using the original IPI model as reference, the added predictive performance for each of the performance indices of the CPH model was more than twice the magnitude of the added predictive value of the NCCN‐IPI. The C‐index was highest for the CPH model (C‐index 0.73), which implies that the order of the risk scores of the CPH model was closer to the true order of the death times than the IPI (0.65), R‐IPI (0.63), and NCCN‐IPI (0.67) risk groups (Table 3). Furthermore, the lowest IBS was achieved with the CPH model (IBS 0.149), suggesting that the predicted survival curves of this model were significantly closer to observed outcome (deaths) than the Kaplan–Meier curves corresponding to the IPI (0.169), R‐IPI (0.172), and NCCN‐IPI (0.164) risk groups (Table 3). The time‐varying AUC plot shows that for each time t, the CPH model was superior to the IPI(‐like) models for discriminating correctly between dead and alive patients. Thus, the CPH model was associated with larger AUC values than the IPI(‐like) indices (Fig. 2). The three performance measures (C‐index, IBS, and AUC) consistently pointed to the CPH model as the most accurate prognostic model, the R‐IPI and IPI models the least accurate models, and the NCCN‐IPI was in‐between the two extremes. The prognostic performance of the IPI and NCCN‐IPI scores (not risk groups) was also inspected. Using raw scores performed better than risk groups but was still inferior to the CPH model (Table 3 and Fig. 2). The performance and performance ranking of the different models was fully reproducible in the Swedish cohort (Table 3 and Fig. 2) and could confirm the prognostic superiority of the CPH model.

**Table 2:** Coefficients of the multivariate Cox proportional hazards model (estimated by using the Danish cohort)

	HR	Lower 95% confidence limit	Upper 95% confidence limit
Age	1.06	1.05	1.07
ECOG performance status			
0	1 (ref)	–	–
I	1.47	1.25	1.72
II	1.97	1.59	2.45
III	2.62	2.04	3.37
IV	3.25	2.32	4.54
Log(LDH)	1.36	1.24	1.50
Number of extranodal sites	1.08	1.02	1.15
Ann Arbor stage			
I	1 (ref)	–	–
II	1.15	0.89	1.48
III	1.44	1.14	1.82
IV	1.37	1.09	1.73

**Table 3:** The integrated Brier score and C‐index of the multivariate Cox proportional hazards (CPH) model, the International Prognostic Index (IPI) grouping, IPI scores, the National Comprehensive Cancer Network (NCCN‐IPI) grouping, NCCN‐IPI score, the revised IPI (R‐IPI) grouping, and CPH models in which one variable used the IPI dichotomization

	Denmark		Sweden	
	IBS	C‐Index	IBS	C‐Index
CPH model	0.149	0.73	0.149	0.73
IPI	0.169	0.65	0.171	0.65
R‐IPI	0.172	0.63	0.172	0.63
NCCN	0.164	0.67	0.164	0.67
IPI‐score	0.168	0.66	0.166	0.66
NCCN‐IPI‐score	0.159	0.69	0.158	0.69

In the Danish data, these were measured using 10‐fold cross validation. In the Swedish data, similar measures were obtained using the CPH model fitted on the Danish data.

The performance, measured by the IBS and C‐index, of the CPH models with one dichotomized variable was essentially on par with the CPH model without dichotomized variables except when dichotomizing age (Table 3).

The PPV of the CPH model was 0.63 in the Danish cohort and 0.62 in the Swedish cohort. This indicates that in the group of patients predicted to have ≥50% risk of dying within 2 years by the CPH model, the percentage of observed events (deaths) was in fact above 50%. In comparison, the PPVs of the high‐risk groups defined by the IPI (DK: 0.48; SE: 0.46), the R‐IPI (0.38; 0.37), and the NCCN‐IPI (0.57; 0.55) indicated that, except for the NCCN‐IPI, the 2‐year survival remained above 50% in the high‐risk groups.

**Figure 3:**
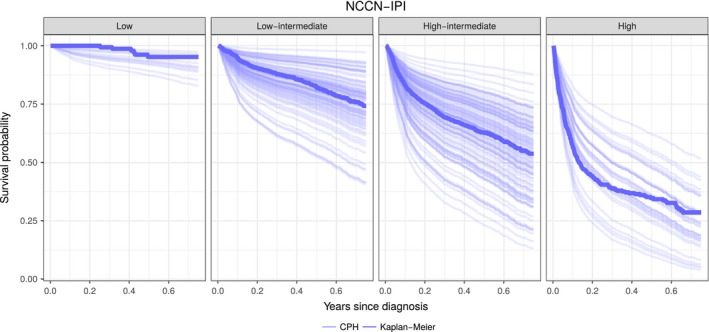
Predicted survival of 250 randomly selected patients in the Danish cohort predicted by the Cox proportional hazards model (CPH) and the Kaplan–Meier method for each National Comprehensive Cancer Network International Prognostic Index (NCCN‐IPI) group. There is a notable spread of predicted survival within each risk group and a certain overlap of estimated survival curves between the risk groups.

The difference between modeling approaches is illustrated by plotting CPH‐specific survival curves within each of the NCCN‐IPI risk group (Fig. 3). For illustrational purposes, the plots only show the survival curves of 250 randomly selected Danish patients. Note the significant spread of survival curves predicted by the CPH model within the NCCN‐IPI‐specific risk groups, emphasizing the potential problems associated with group‐based outcome predictions (IPI, R‐IPI, and NCCN‐IPI) for individual patients.

Since models similar to the CPH model are characterized by higher complexity, precluding use without dedicated web‐interfaces, we developed lymphomapredictor.org. On this page, users can test the CPH model versus the conventional IPI, R‐IPI, and NCCN‐IPI models. The website was programmed using the “shiny” package and allows users to enter patient characteristics 23. Furthermore, to facilitate interpretation, we also provide the survival of an age‐gender matched background population of the Nordic countries 24.

## Discussion

Using population‐based lymphoma databases, we were able to evaluate and compare the original IPI to recently proposed variations, the R‐IPI and the NCCN‐IPI. In addition, and more importantly, we were able to show a substantial gain in prognostic accuracy by assuming linearity for the effects of continuous variables and using survival probability to predict outcome instead of dichotomized variables and predefined risk groups.

Our results indicate that the IPI outperformed the R‐IPI with small margins for each of the summary measures presented. Thus, simply regrouping the same risk factors into other risk groups did not lead to an increased predictive accuracy of the R‐IPI over the IPI. However, we did see a significant benefit of using incremental scores for increasing values of continuous risk factors as proposed in the NCCN‐IPI 9. Using this approach, the NCCN‐IPI was capable of providing better risk stratification than both the R‐IPI and IPI, which underscores that substantial improvements in predictive accuracy are obtained by models that include more than two categories for risk factors. Furthermore, the performance of both the IPI and NCCN‐IPI can be improved using the scores instead of the risk groups to rank patients. Taking this approach a step further, we developed a CPH model that was based on the exact same variables as the IPI, but without dichotomization or the use of a few risk groups as outcome. This model assigned increased risk for each incremental values of age, Ann Arbor stage, normalized LDH ratio, number of extranodal sites, and ECOG performance score. When this model was tested against the NCCN‐IPI, IPI, and R‐IPI using appropriate statistical performance tests such as the Brier score, time‐varying AUC, and C‐index, the CPH model outperformed the other models. This finding clearly indicates that current models fail to exploit the full potential of available clinical data. The performance tests yielded similar results in the Swedish DLBCL population, which indicates that these results are generalizable to other countries.

To investigate the causes of loss in predictive power also models including one dichotomized variable, dichotomized as in the IPI‐score, were fitted. The performance measures indicated that dichotomizing age leads to large losses in predictive performance. The dichotomization of the other variables did not cause major losses in predictive performance. This highlights the importance of age as a prognostic factor for overall survival. Given that dichotomization only explained part of the information loss in the IPI‐like models, we can conclude that also assigning the dichotomized variables the same weight in the final risk scores as well as grouping patients into risk groups instead of providing continuous risk scores cause large losses in prognostic performance.

Meaningful clinical risk scores should identify patients likely to fail standard therapy. In Zhou et al. focus was on finding a patient subgroup with a 5‐year survival below 50% 9. Since long‐term survival is strongly influenced by deaths due to old‐age, we focused on discovering a subgroup with a 2‐year, instead of 5‐year, OS below 50%. The relatively high PPV for patients predicted to have a 2‐year OS below 50% confirmed that the CPH model was highly capable of identifying patients with poor survival and for whom experimental therapies or alternative treatment approached may be relevant. The 2‐year OS of high‐risk groups according to the R‐IPI and IPI and associated PPV showed that these widely used models were unable to discover a meaningful high‐risk group unlike the NCCN‐IPI and the CPH model.

Within the risk groups defined by the best conventional model, the NCCN‐IPI, we observed a large spread in the predicted survival (Fig. 3). This visualizes the interpretational problems clinicians have to deal with when group‐based risk models are used for individual patient assessment. The CPH model became closer individualized outcome prediction by not forcing patients into few predefined risk groups. Furthermore, there was substantial overlap between survival predictions of patients in adjacent NCCN‐IPI risk groups (Fig. 3). Both these observations indicated that risk groups based on dichotomized covariates led to a substantial loss of prognostic information.

Maurer et al. (2016) reported similar effects using event‐free survival (EFS) at 24 months as endpoint. The EFS at 24 months was chosen since, in other studies, DLBCL survival normalized relative to that of a matched background population at this milestone time point 25, 26, 27. Maurer et al. (2016) used a logistic regression model with continuous variables to calculate the probability of EFS at 24 months and compared the regression model to the IPI model 25. Consistent with our finding, a large spread of predicted probabilities within each of the IPI groups was noted 25. However, the logistic regression model also included clinical variables that are not part of the IPI (bulky disease, absolute lymphocyte count, and sex). Furthermore, IPI was developed as a general prognostic model and not for predicting 2‐year EFS 6. Thus, differences in predictive performance of the logistic regression model presented by the authors and the original IPI are not explained only by avoiding dichotomizing the variables and using risk groups instead of continuous risk scores.

The purpose of this study was to compare the predictive performance of simple clinical scores with a model using the same covariates. To keep the exposition simple, only a model with linear effects was reported (the CPH model). Tests with nonlinear covariate effects, in particular spline effects, did not lead to noticeable improvements in the prognostic performance (data not shown). We do not expect the presented CPH model to make IPI‐like models obsolete, but our intention was to show how simple clinical data used in slightly more sophisticated ways can improve predictive accuracy substantially and be of high value even in the era of molecular genetics. This is relevant, in particular, because testing for molecular high‐risk disease may not be available to all clinicians in all parts of the world. Finally, while molecular genetics is becoming of utmost importance for risk profiling in DLBCL, we should be aware that clinical and genetic risk factors frequently overlap; double/triple hit DLBCL cases are often featured by disseminated extranodal involvement, which in itself is associated with poorer outcomes 28, 29, 30.

Further refinements of clinical risk models could be achieved by increasing the number of clinical variables included. For example, albumin concentration and the site of extranodal involvement may add prognostic value 8, 31. Additionally, refinements of the IPI and NCCN‐IPI have led to adjusted risk scores for the elderly 6, 32, which indicate the importance of interaction effects. Furthermore, the effect of age and other continuous variables is likely to be nonlinear and the proportional hazards assumption of the CPH model is likely violated 9, 33. However, in predictive modeling, unlike in classical inference about the covariate effects, model selection by extensive assumption checking can be fairly replaced using the generalization/prediction error as model selection criterion 34. Future research will focus on using different model formulations and more variables to further enhance outcome prediction from clinical data. To accomplish this task, different flexible modeling approaches such as spline models and random survival forests may be useful 35, 36. The main drawback of complex survival models is a need for dedicated software for implementation in routine clinical setting. However, since most physicians have mobile devices available this limitation is easily overcome today. Our CPH model was implemented in the form of an interactive web page (lymphomapredictor.org), where clinicians can test it against conventional models.

Similar to IPI‐like models, the CPH model classifies a relatively large proportion of patients to be at intermediate risk of dying within 2 years. Even though future modeling efforts might lead to further model improvements, patients at intermediate risk (≈50%) will likely still exist. These groups are clinically challenging, as risk estimates around 50% equal tossing a coin. However, uncertainties like these arise, at least in part, from many unknown factors that may not be related to DLBCL. These include deaths from other causes, which are particularly relevant in a disease of old age like DLBCL. Using cause‐specific survival and disease‐free survival as end‐points may avoid this issue, but we were not able to provide these end‐point due to the structure of databases used in the study. Other reasons may be the fact that use of baseline information will not be able to predict future events with 100% accuracy. Patients’ decisions regarding treatment and serious adverse events and complications to treatments between diagnosis and the time of interest are factors that influence survival but cannot be captured by baseline clinicopathologic features.

A drawback of the used registers is the risk of incorrect disease classification. In particular, primary mediastinal B‐cell lymphoma (PMBCL) cases could have been registered as DLBCL to some extent. The outlook for PMBCL patients tends to be better than in DLBCL, but given the rarity of PMBCL as compared to DLBCL potential biases arising from this small group are likely to be insignificant 32.

In conclusion, we hope that future developments in statistics and machine learning will allow exhaustive use of the information available in our large clinical databases and pave the path toward personalized medicine based on a combination of clinical data and molecular genetics rather than the latter alone.

## Conflict of Interest

None declared.

## Supporting information


**Figure S1.** Flowchart describing the inclussion criteria.
