# Corrigendum

**DOI:** 10.1002/cam4.1482

**Published:** 2018-04-22

Simplicity at the cost of predictive accuracy in diffuse large B‐cell lymphoma: a critical assessment of the R‐IPI, IPI, and NCCN‐IPI

Jorne Biccler, Sandra Eloranta, Peter de Nully Brown, Henrik Frederiksen, Mats Jerkeman, Karin E. Smedby, Martin Bøgsted & Tarec C. El‐Galaly.

Cancer Medicine 2018; 7: 114–122.

The published version of Table 3 for this article was incorrect. The correct Table 3 and its caption are shown below:

**Table 3:** The integrated Brier score (IBS) and C‐index of the multivariate Cox proportional hazards (CPH) model, the International Prognostic Index (IPI) grouping, IPI scores, the National Comprehensive Cancer Network (NCCN‐IPI) grouping, NCCN‐IPI score, the revised IPI (R‐IPI) grouping, and CPH models in which one variable used the IPI dichotomization

	Denmark		Sweden	
	IBS	C‐Index	IBS	C‐Index
CPH model	0.149	0.73	0.149	0.73
IPI	0.169	0.65	0.171	0.65
R‐IPI	0.172	0.63	0.172	0.63
NCCN	0.164	0.67	0.164	0.67
IPI score	0.168	0.66	0.166	0.66
NCCN‐IPI score	0.159	0.69	0.158	0.69
CPH model, dichotomized age	0.160	0.70	0.156	0.71
CPH model, dichotomized LDH	0.150	0.73	0.149	0.73
CPH model, dichotomized number of extranodal sites	0.150	0.73	0.149	0.73
CPH model, grouped Ann Arbor stage	0.149	0.74	0.148	0.73
CPH model, grouped ECOG performance status	0.150	0.73	0.151	0.72

The corresponding author apologizes for this error.

Biccler, J., S. Eloranta, P. de Nully Brown, H. Frederiksen, M. Jerkeman, K. E. Smedby, M. Bøgsted and T. C. El‐Galaly. 2018. Simplicity at the cost of predictive accuracy in diffuse large B‐cell lymphoma: a critical assessment of the R‐IPI, IPI, and NCCN‐IPI. Cancer Med. 7:114–122.

