# Supplementary figures and images for: Simplicity at the cost of predictive accuracy in diffuse large B‐cell lymphoma: a critical assessment of the R‐IPI, IPI, and NCCN‐IPI
Source: Cancer Med. 2017 Dec 13;7(1):114–22. doi: 10.1002/cam4.1271 (PMC5773951; doi:10.1002/cam4.1271)

## SUPPLEMENTARY MATERIAL

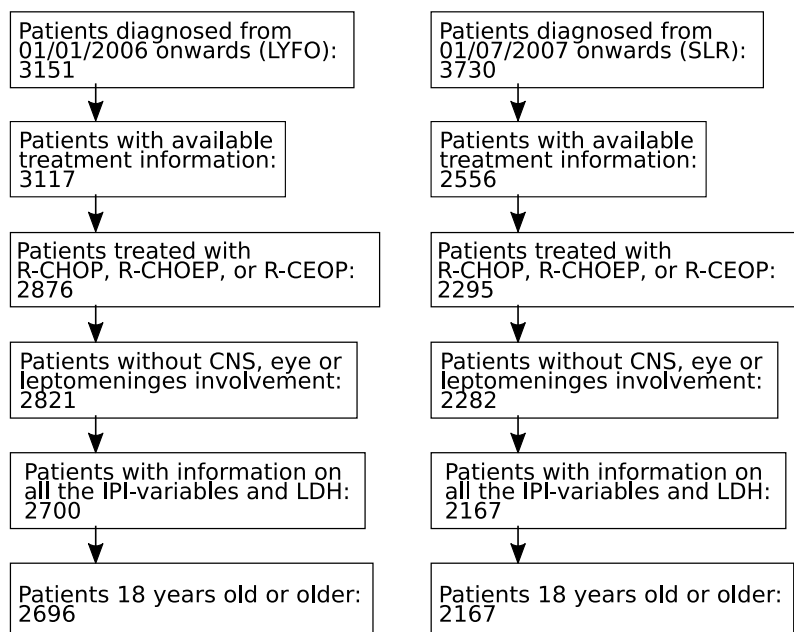

Figure 1. Flow-chart describing the data-cleaning process.

Supplement: Supplementary file 1 — Figure S1. Flowchart describing the inclussion criteria. [file CAM4-7-114-s001.pdf]
